# Where Did You Hear About Us?: Examining How Referral Sources Impact Recruitment and Retention Within a Behavioral Parent Training Program

**DOI:** 10.1002/jcop.70019

**Published:** 2025-06-03

**Authors:** Abigail Peskin, William Andrew Rothenberg, Camille Perez, Cindy Sobalvarro, Eileen Davis, Elana Mansoor, Jason Jent, Dainelys Garcia

**Affiliations:** ^1^ University of Miami Miami Florida USA

**Keywords:** behavior therapy, parent training, recruitment, referral, retention

## Abstract

Behavior problems in young children, especially among families from disadvantaged backgrounds (e.g., facing greater risk of poverty, social exclusion, discrimination, and violence), often result in referrals to mental health clinics. However, low‐income families from historically marginalized racial and ethnic backgrounds disproportionately experience barriers to accessing, engaging, and completing treatment. This study examined the recruitment and referral network of a parenting program providing Parent‐Child Interaction Therapy (PCIT) in a large urban academic medical center and affiliated community‐embedded clinics, as well as the impact of recruitment/referral sources on screening paperwork completion, intake attendance, and treatment completion. Data from 2510 families referred between 2018 and 2022 were analyzed, considering demographic factors and recruitment adaptations during COVID‐19. Referral sources included but were not limited to community agencies, social media, and healthcare providers. Logistic regression analyses determined the likelihood of completing the screening paperwork, attending intake, and completing treatment based on recruitment/referral sources. Every recruitment/referral source increased the likelihood of screening paperwork completion (except community outreach). Every source increased the likelihood of attending intake (except previously enrolled families). Treatment completion was significantly more likely for those referred from pediatricians, friends, behavioral health, and Google. After COVID‐19 (post March 2020), families were more likely to complete the screening paperwork, attend intake, and complete treatment compared to families screened before COVID‐19. Effective recruitment and retention strategies are crucial for engaging families in mental health services. Findings emphasize the role of community and healthcare providers, word‐of‐mouth, and Google and the benefits of telehealth (indicated by post‐COVID‐19 results), in improving treatment access and retention, highlighting the need for flexible service delivery methods.

Behavior problems in young children are prevalent and have been shown to be the most common reason for referral to mental health clinics (Breitenstein et al. 2009). Disadvantaged (e.g., facing greater risk of poverty, social exclusion, discrimination, and violence) and historically marginalized (e.g., not prioritized for receiving treatment, not easily able to access treatment) children and their families are not only more likely to report behavior problems, but also less likely to access, engage in, and benefit from evidence‐based parenting interventions‐ proven to be highly effective in addressing child behavior problems (Barnett et al. 2020; Chacko et al. 2016; Coker et al. 2009). The ability to effectively recruit and retain historically disadvantaged and marginalized families in parenting interventions is critical to addressing disparities related to access to and engagement in these programs.

A range of individual, neighborhood, and structural barriers contribute to recruitment/referral and retention challenges. Recruitment/referral here refers to getting families connected with applicable resources, and retention refers to keeping families in treatment once they have started. The challenges with both recruitment and retention tend to be exacerbated among disadvantaged and historically marginalized families, including historical misuse of research to exploit, rather than partner with, communities (i.e., leading to mistrust of medical providers; Coker et al. 2009; Hughes et al. 2015; Yancey et al. 2006), difficulty obtaining reliable transportation to services (Koerting et al. 2013), distance to the service location (Bradshaw et al. 2020), difficulty accessing treatment in the caregivers' preferred language, and difficulty obtaining child care to attend treatment (Smokowski et al. 2018).

## 1 Recruitment and Retention

In an effort to address recruitment and retention barriers, multiple strategies have been identified that emphasize community connections and cultural sensitivity (Reidy et al. 2012). Specifically, when comparing various strategies for recruiting Hispanic families into parenting programs (e.g., providing cash incentives for attendance, offering free childcare, employing bilingual staff and facilitators, translating recruitment materials to Spanish), Reidy et al. (2012) found community liaison partnerships to be one of the three most important recruitment strategies (Metayer et al. 2018; Reidy et al. 2012; Smokowski et al. 2018). In a community liaison partnership, a community leader/project “champion” serves as a liaison between the researcher/clinician and the families. Research also suggests that recruitment and retention strategies for disadvantaged and marginalized families may differ across groups. For example, Metayer et al. (2018) found that media announcements were more effective than connections with other community organizations for recruiting Haitian families.

While several recruitment strategies have been developed based on cultural responsiveness and community connections, other strategies have focused on more traditional direct marketing approaches. These direct approaches include face‐to‐face recruitment (Adams et al. 2017), targeted mailings (Richmond et al. 2013; Yancey et al. 2006), or a combination of in‐person delivered and mailed flyers (Sharpe et al. 2021), all of which have been shown to be effective at enrolling families, particularly when financial compensation is included (Pescud et al. 2015) or when followed by a recruitment phone call (Abraczinskas et al. 2021). Since the onset of COVID‐19 and the rise of telehealth services, research such suggests social media has become a more effective recruitment strategy, with organic Facebook posts and engagement being identified as the most effective approach to recruiting participants for treatment (Archer‐Kuhn et al. 2022).

Though existing research provides promising leads in determining what types of recruitment/referral sources (e.g., Facebook, flyers, word‐of‐mouth) lead to family engagement and retention in behavioral health services, this body of literature is small, and few studies have systematically examined a range of recruitment/referral sources (e.g., pediatric medical clinics, social media, allied health professionals, etc.). In addition, it seems likely that the relative effectiveness of referral/recruitment sources may differ for different groups. For example, Latino families may be more likely to connect with services when referred by community agencies that offer support in other ways, or by word‐of‐mouth (Zamora et al. 2016). Word‐of‐mouth often emerges as one of the most successful referral/recruitment sources (Goin‐Kochel et al. 2024), and has also been demonstrated as one of the more highly effective recruitment sources for African American participants (Horn et al. 2020; Stahlschmidt et al. 2013) in some studies, while others have seen word‐of‐mouth be less effective than targeted mailing or flyers posted in the community (Richmond et al. 2013). Other studies have found that referral source has no impact on a family's likelihood of enrolling in and completing a parenting program (Heidari et al. 2018). Although it is impossible for a community clinic to measure how widely a providers' referral/recruitment sources “spread the word” in their networks (because it is difficult to measure how many people were told about the services but did not contact that clinic), it is vital to know which referral sources make it most likely that participants are motivated enough to contact the clinic and complete treatment. That is our focus here: identifying referral sources that make it most likely participants complete screening paperwork to gain access to treatment, attend the first session, and complete treatment.

Literature examining the barriers to recruiting historically underrepresented groups (e.g., ethnic minority, low income) may serve to illuminate some of the reasons why certain referral sources are effective and others are not. For example, some participants choose not to participate in research due to mistrust of academic institutions (often as a result of historic abuse and exploitation; Yancey et al. 2006). Some researchers may overcome this barrier to recruitment by establishing relationships with otherwise trusted professionals (e.g., pediatricians) and community‐based organizations (Schoeppe et al. 2014), who then become partners for participant recruitment. Recruitment efforts that lacked cultural competence have also been noted as a reason why parents do not enroll in research studies (Coker et al. 2009). This includes both a lack of understanding of participants' values as well as lack of recruitment in areas of communities where families gather. These barriers are sometimes overcome by partnering with community liaisons and services that exist within the community (e.g., places of worship, day care centers). Specifically, community health workers (CHWs) can help families overcome barriers to accessing mental health treatment because they are embedded within the community, allowing them to effectively reach difficult‐to‐access populations including minorities and address disparities in access (Gustafson et al. 2018). Additionally, CHWs can serve as community advocates who liaise between institutions and community members, while their peer status helps them develop more trusting relationships within the community. Additionally, often one of the most effective ways to recruit participants from historically underrepresented groups is to work with a few families in a culturally sensitive manner, to establish trust through a few families who will then tell others in their communities about the services offered. This word‐of‐mouth allows people to hear from those they trust and thus often increases their likelihood of connecting with services (Goin‐Kochel et al. 2024; Horn et al. 2020; Stahlschmidt et al. 2013).

Social proximity theory may serve as another explanation for the relative effectiveness of certain referral/recruitment sources over others. Specifically, social proximity theory posits that families may be more likely to seek, refer, or accept services from others who are socially close to them—such as friends, family, neighbors, or culturally similar peers—due to trust, familiarity, and shared values (McPherson et al. 2001; Valente 2010). Indeed, several aspects of this theory support that families will be more likely to seek services from those trusted by those in their networks. Families are more likely to seek advice from those they trust, and to follow that advice from those trusted others. Providers are also more likely to refer to other providers they trust (Valente 2010). This trust between providers and other referral/recruitment sources means that connections are more likely between culturally similar providers and more informal referral sources of recruitment like word‐of‐mouth (Valente 2010).

Examining and systematically comparing the effectiveness of different recruitment/referral sources in engaging families in treatment is especially important because most behavioral health providers do not recruit from just one referral source, but several. Literature comparing the effectiveness of these disparate recruitment/referral sources is needed. Community behavioral health programs often have limited employee time and marketing resources available, and therefore need to allocate these limited resources to those recruitment/referral sources that are most effective in recruiting families who will be engaged and retained in services. This is especially true for grant, state, or federally funded clinics that attempt to provide reduced cost or free services to families who otherwise might not be able to obtain treatment, like ours. For such clinics, it is especially crucial to recruit and retain clients to ensure grant and contract deliverables are met to ensure clinics can continue to offer low‐cost services.

The present study closes this gap by systematically investigating recruitment and retention of families in behavioral health services from a range of recruitment/referral sources, including pediatric primary care, behavioral health, Google, early intervention, school services, community outreach, allied health professionals, disability advocacy organizations, wraparound neighborhood services, social media, word‐of‐mouth, schools, and physician specialists.

## 2 COVID‐19

The landscape of mental health services changed drastically in the wake of the COVID‐19 pandemic, when therapists rapidly transitioned to online delivery of services (Garcia et al. 2021; Peskin et al. 2024; Phillips et al. 2021; Pierce et al. 2021). What before the pandemic were regular sources of referrals (e.g., schools, doctors' offices, community partner agencies, etc.), either closed for months or transitioned virtually. These changes necessitated a pivot in recruitment and referral processes for mental health services, at a time when the need for mental health care was increasing precipitously (O'Connor et al. 2020; Racine et al. 2021). Though the pandemic has ended, its impact on recruitment and referral processes for mental health services likely continues to influence present‐day strategies (Archer‐Kuhn et al. 2022). The current study investigates the effectiveness of various recruitment/referral sources in recruiting and retaining predominantly disadvantaged and marginalized families, considering the changes brought about by the pandemic. The objective is to analyze the referral network of a university‐based PCIT clinic. Specifically, this study aims to determine which recruitment/referral sources not only generate a high volume of referrals but also yield participants who (a) complete screening paperwork, (b) attend intake, and (c) complete treatment.

## 3 Methods

### 3.1 Participants

**Figure 1:**
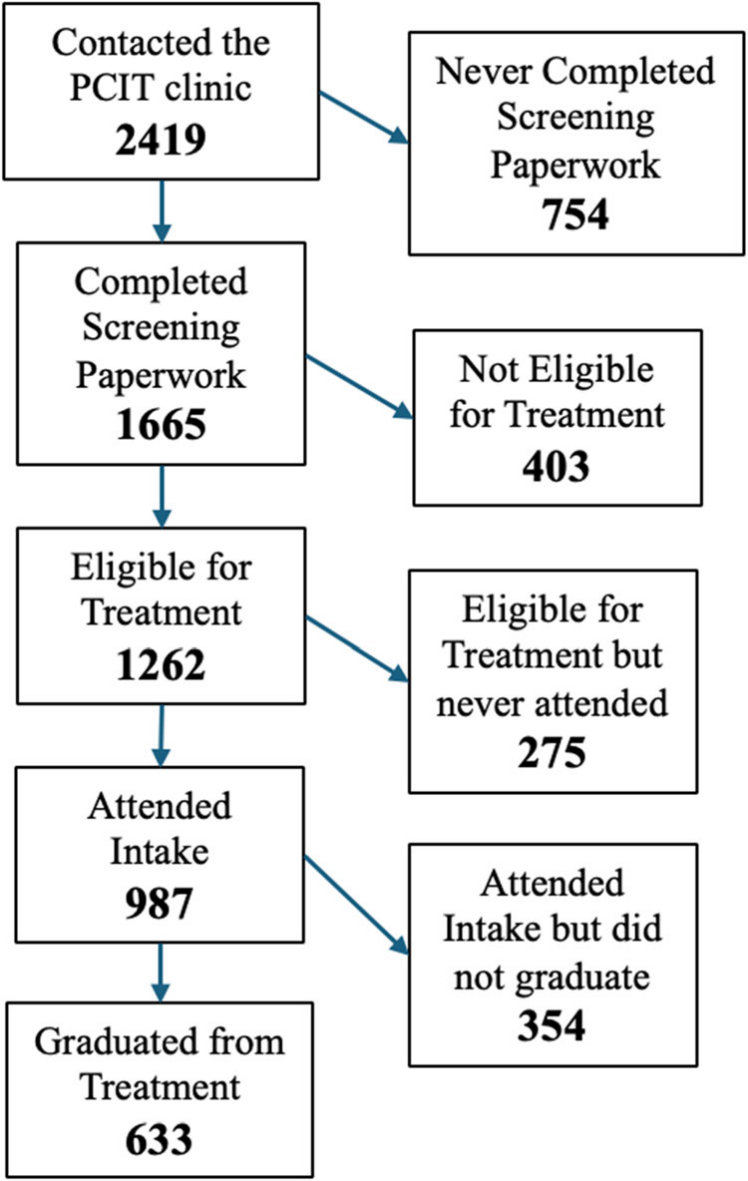
Flowchart of families from initial clinical contact to treatment completion.

Participants for this study included 2407 families that were referred to PCIT and completed an initial eligibility screening between 2018 and 2022. Children screened were between the ages 1 and 12 (*M* = 4.33, SD = 1.75). The number of participants for some demographic categories fluctuates because, although child age, caregiver level of education and preferred language were gathered at screening, other more detailed demographic variables were not gathered until intake. For those who completed intake paperwork and provided more detailed information, demographics are included in Table 1. The flow of participant numbers from screening to intake to graduation is depicted in Figure 1.

**Table 1:** Demographic information for families who completed intake paperwork.

Caregiver ethnicity	Hispanic	Non‐Hispanic
	793	318
Caregiver race		
American Indian or Alaskan	5	1
Asian	1	8
African American	11	89
White	708	145
Other	13	13
Multiracial	49	38
Haitian or English‐Speaking Caribbean	0	23
Language of PCIT delivery		
English	476	305
Spanish	304	12
English and Spanish	13	1

Families who completed the eligibility screening during COVID‐19 did not differ from those before the pandemic with regard to child ethnicity or age, although there were differences in race and language. Specifically, White children were significantly *more likely* (pre‐COVID‐19: 73.81%; COVID‐19: 80.48%; *χ* ^2^ [1, 1088] = 7.184, *p* = 0.005) and African American children significantly *less* likely (pre‐COVID‐19: 11.17%; COVID‐19: 7.20%; *χ* ^*2*^ [1, 1088] = 5.153, *p* = 0.015) to receive PCIT during the pandemic. Families receiving services during the pandemic were also significantly more likely to speak Spanish (32.22%) than those prior (27.40%; *χ* ^*2*^ [3, 1726] = 38.916, *p* < 0.001).

Study inclusion criteria consisted of (a) the child being 2–8 years old at the time of treatment (children under 2 were occasionally screened if they would soon turn 2, at which point services would begin), or up to 12 if there was a history of involvement with child protective services (e.g., see Chaffin et al. for information on PCIT with child welfare population), (b) the primary caregiver being fluent in English, Spanish, or Creole, (c) the family living in the specific county in South Florida where the clinic was located (i.e., a requirement from the grant funder), (d) elevated child disruptive behavior on the Eyberg Child Behavior Inventory Intensity Scale (ECBI; Intensity Raw Score ≥ 131; Eyberg and Pincus 1999) or on the Externalizing Problems subscales or composite of the Behavior Assessment System for Children, Third Edition (BASC‐3; T‐Score ≥ 60; Reynolds and Kamphaus 2015), or a history of involvement with child protective services. Families who did not meet inclusion criteria were given referrals and resources. Institutional Review Board (IRB) approval was obtained from the university and all participants who agreed to be in the study signed an informed consent. All study procedures were conducted in accordance with the ethical standards of the IRB.

### 3.2 Study Design and Procedures

#### 3.2.1 Screening

Families were screened for services through a phone eligibility screening, which included providing verbal consent and completing a background form and behavioral questionnaires in English or Spanish. If inclusion criteria were met, families completed the intake appointment before starting treatment.

During the study recruitment period, the PCIT program introduced a series of screening enhancement strategies to improve family participation. These included streamlining phone screening procedures with systematic follow‐up calls, shortening the intake form duration (from 40 to 10 min), transitioning all screening paperwork to the online data capture platform REDCap (Harris et al. 2009), and providing a widely distributed public survey link on the website to eliminate the need for initial phone screening before completing paperwork.

#### 3.2.2 Treatment

Following the screening and intake process, families began Parent‐Child Interaction Therapy (PCIT; Eyberg and Funderburk 2011), a parenting program involving weekly evaluations of child behavior and parenting skills taught to caregivers across two phases: Child‐Directed Interaction (CDI) and Parent‐Directed Interaction (PDI), over the course of 18 weeks. Therapists provided coaching to caregivers on their parenting skills, either through a wireless headset behind a one‐way mirror (for in‐person sessions) or via secure videoconferencing (for telehealth sessions). The PCIT program examined in the current study was based in a large urban academic medical center with affiliated community‐embedded clinics (i.e., clinics embedded in underserved and low‐income neighborhoods within trusted community agencies). Families receive PCIT services in one of six clinics or telehealth (I‐PCIT), although telehealth services were less prevalent before COVID‐19 and transitioned fully to telehealth after March 16, 2020. Two treatment sites were located in academic centers, while the remaining four clinics were embedded within local communities in a large metropolitan area in the Southeast United States.

#### 3.2.3 Recruitment

Following the transition to delivering services exclusively via telehealth in response to COVID‐19, a recruitment team was created with select PCIT trained therapists (masters and doctoral‐level) and members of the screening and data team, who met monthly to discuss recruitment strategies. The team identified new avenues for optimizing recruitment and for deepening relationships with current recruitment/referral sources, including sending thank you letters or emails following receipt of referrals, creating systems for warm handoffs from community partners, and distributing written and electronic flyers. Warm handoffs included recruitment/referral partners calling the PCIT program together with the family, walking the family to meet the program (if they were located in the same building), sending the family's information directly to the screening and data team (with the family's consent), and following up with the family after referral to ensure that the connection was effectively made. Recruitment/referral sources are described in Table 2, as well as the strategies by which the recruitment team established and maintained relationships with community partners. During the first screening phone call and screening paperwork, families were asked about how they learned about the PCIT services/clinic. This response was used as the official recruitment/referral source for purposes of the analyses in this study. Table 2 indicates whether these recruitment/referral sources were colocated with the PCIT clinic, and whether the referring provider switched from in‐person to telehealth services during COVID‐19. For coding purposes, insurance companies were used as the reference variable for dummy coding referral sources.

**Table 2:** Recruitment/referral sources for PCIT program and the strategy used for referring between the program and PCIT.

	Operational definition	Strategy of referral	Colocated with one of the PCIT clinics	Provider switched from mostly in‐person to some or all telehealth during COVID‐19
Pediatric primary care	Pediatricians and other medical personnel that families seek for primary care	Families were referred directly from the medical professional, and for pediatricians located in the same university as the clinic, occasionally referrals were made via email directly from the pediatrician	X	X
Word of mouth	Families who have previously participated in PCIT refer their friends	Not a source actively recruited—families gave their friends the clinic information and their friends contacted the clinic	N/A	N/A
Behavioral health	Mental health providers in the community including: − Therapy providers who offer services to different populations (e.g., anxiety, older kids) − Psychologists completing diagnostic assessments who subsequently refer for therapy services − One‐time preventative mental health workshops that refer for more intensive services	− PCIT referred for testing and for initial preventative workshops − Behavioral health providers referred to PCIT		X
Google	Families used a search engine to find clinics for managing child behavior. This clinic showed up on the first page of search results on Google. Results were available in English and Spanish.	N/A		
Physician specialists	All those with MD or DO who were not pediatricians. This category often included neurologists but also included psychiatrists and other specialty physicians.	− PCIT referred to these specialists as needed, but rarely − Physician specialists referred to PCIT by providing families with the information to contact the clinic	X	
Early intervention	Part C of IDEA in South Florida—For children 0‐3 years, developmental assessment and intervention for children at‐risk for developmental disabilities	Colocated with PCIT in the University setting. Referred by warm handoff; an early intervention staff member contacts a PCIT staff member with the family present, and on‐site PCIT staff then explain the program and answer families' questions, either in‐person or over the phone.	X	X
School‐based services	Public and private schools and afterschool programs in the community	PCIT program contacted nearby schools (e.g., guidance counselors) and provided information about PCIT. A follow‐up email would then be sent with an attached flier to share with families. Some schools put brochures in the office area for families to learn about PCIT.		X
Community outreach	− Community Fairs: Clinic team employees (e.g., screening staff, clinical supervisors, therapists) set up booths at community fairs and other events. These events were held by the grant‐funder for PCIT (serving over 10,000 visitors) and other community organizations. − Brochure: the PCIT program distributed flyers widely throughout the community, to post on bulletin boards in community centers and libraries, and for families to pick up in doctor's waiting rooms − Talk/Presentation: Occasionally the PCIT team presented at community events to educate families about the availability of their services	− Information was provided for families to contact the clinic if they needed services − Sometimes a sign‐up sheet was provided for the event where families could provide their information, and then the clinic staff would follow up with them by phone following the event		
Allied health professionals	− Speech‐Language Pathologists, Occupational Therapists, and Physical Therapists in the community − A grant‐funded program for kids 0‐5 with mild developmental delays	− Allied Health Professionals referred families by sharing PCIT's clinic and contact information. Family contacted PCIT. − Grant‐funded program sometimes directly referred by emailing the family's information to the PCIT screening team with family consent	X	
Disability advocacy organizations	− Autism Community Services (ACS), a state‐wide non‐profit offering free programs (e.g., support groups) and connection to resources in the community for children and adults with autism and other related disabilities, and their families. − Parent Advocacy, established in 1986, helps families obtain school‐based services for their children (e.g., IEP and 504 services), and improves family functioning and parenting practices.	− Individual ACS staff referred families to PCIT when they needed behavioral services − ACS advertised PCIT via their weekly emailed newsletter − PA and PCIT referred families to one another due to complementary services; children with disruptive behavior often need support in both the school system and the clinic. Referrals between PCIT and PA lacked a formalized process, and mainly took place by providing families with contact information for the other program.		X
Wraparound neighborhood services	A satellite PCIT clinic was embedded within a wraparound community center in a primarily Hispanic, Spanish‐speaking neighborhood. The community center near downtown was established in 2010, and connects adults, children, and families with resources in the community, including food distribution, registers children and provides advocacy in schools, recommends parenting classes, and strengthens communities. PCIT connected with them in 2014 as part of an effort to train family support workers (who provided advocacy and case management services) in learning and implementing the concepts of PCIT with families enrolled in both agencies in their homes (Barnett et al. 2016; Davis et al. 2022; Garcia et al. 2023). The Wraparound Community Services referral source included additional supports to help families make it to intake and complete treatment, a component that was not consistently present in other referral sources utilized by the clinic.	Families receiving services at this wraparound clinic were referred directly to the colocated PCIT services. Community health workers at the location helped families to complete intake paperwork.	X	
Social media	− Clinic Facebook page est. in 2018. − Posts approximately twice per week about child behavior/development and community events − Posts shared with local Facebook groups − Instagram account est. 2020 − Posts twice weekly with books about social‐emotional topics and child behavior management strategies − Engagement with accounts of other local child organizations − Hashtags focused on local kids/families	Families rarely contacted the clinic directly on social media, but were able to access a public link there that led to the PCIT program's screening questionnaire.		
Previously enrolled	− Occasionally families who had previously completed PCIT contacted the clinic to re‐enroll—either to complete again with the same child, or to enroll with a different child or different caregiver	Families either contacted the general clinic line or directly reached out to the therapist they had seen previously.		

#### 3.2.4 Measures

For screening paperwork, families completed a universal intake questionnaire (used throughout the university where the program was based), which consisted of information about caregiver concerns about the child, family information (e.g., who lives in the home, ages, relationship to the child, family significant medical and psychological history), and school information and child birth information (e.g., birth weight, complications at birth, etc.). At that time, families also completed information about their weekly availability to attend PCIT sessions. This initial screening information was available for all families who completed the screening paperwork, but not for those who merely contacted the clinic and never completed the paperwork.

At intake, families completed additional measures to assess child behavior (e.g., ECBI, BASC‐3), caregiver stress (e.g., Parent Stress Survey), child development (e.g., Ages and Stages Questionnaires), and other areas of child development (e.g., child sleep, toileting, eating; caregiver depression and anxiety). At intake families also completed more detailed information about themselves, including their income level, caregiver and child ethnicity, and caregiver and child race. These variables are therefore only available for caregivers who attended intake *and* completed all the intake paperwork. Intake paperwork was assigned following the first intake session, so if families discontinued after the first session the intake paperwork would not be completed.

##### 3.2.4.1 Defining the Time Points

Table 3 presents the number of families for each referral source who: (a) completed eligibility screening, (b) completed screening paperwork, (c) screened as eligible on assessments, (d) attended intake and (e) completed treatment. Those who completed the eligibility screening (a) are those who called the PCIT program to express interest in PCIT, answered a few brief questions to determine that they were in the county the program was offered, had concerns about child behavior, and had a child the appropriate age for PCIT. Those who completed screening paperwork (b) filled out all of the paperwork sent (either via email or RedCap) to determine whether child behaviors were severe enough to qualify for PCIT, and to collect more information about school, family, etc. (see above for more details). Those who screened as eligible on assessments (c) reported clinically elevated child behavioral concerns and were thus put on a waiting list for PCIT. Those who attended intake (d) were marked as having attended their first PCIT session. Those who graduated treatment (e) were marked as completing 18 weeks of PCIT.

**Table 3:** Recruitment/Referral sources from screening to treatment completion.

Referral Source	Number who contacted	Completed screening paperwork	Eligible for treatment	Attended intake	Completed treatment
Pediatric primary care	614	450	341	260	175
Word‐of‐mouth	284	213	162	127	86
Behavioral health	276	203	143	131	93
Google	198	139	101	80	53
Physician specialists	201	136	107	84	44
Early intervention	136	106	79	59	35
School‐based services	132	82	63	50	30
Community outreach	130	64	50	37	24
Allied health professionals	88	64	47	31	19
Disability advocacy organizations	84	63	41	33	20
Wraparound neighborhood services	65	58	55	39	18
Social media	52	42	37	26	18
Previously enrolled	24	20	13	9	6
Total	2317	1658	1257	981	630

### 3.3 Data Analysis

Outcomes measured for analysis included: whether a family (a) completed their screening paperwork, (b) attended intake, and (c) completed treatment. These outcome variables were compared via chi square analyses for family demographic variables of child race, ethnicity, and language of treatment, and via *t* test for child age. Demographic variables that emerged as significant were entered as covariates in subsequent analyses for their corresponding outcomes. Separate logistic regression analyses were then completed for all three outcomes, the dependent variables of (a) completion of screening paperwork, (b) intake attendance, and (c) treatment completion (i.e., completing all requirements for PCIT treatment and graduating; all coded 0 = no, 1 = yes), with the dummy coded sources of referral as predictors. COVID‐19 was also entered as a predictor into each regression, with 0 = pre‐COVID‐19 (i.e., completed treatment before March 2020) and 1 = COVID‐19 (i.e., completed treatment after March 2020).

## 4 Results

### 4.1 Summary of Initial Descriptive Findings

For each recruitment/referral source, Table 2 presents the number of families who: (a) completed eligibility screening, (b) completed screening paperwork, (c) screened as eligible on assessments, (d) attended intake and (e) completed treatment. Screening completion, intake attendance, and treatment completion were all separately examined across demographic variables (e.g., language of treatment, caregiver race, ethnicity) using *t* tests (i.e., for child age) or *χ* ^2^ analyses (i.e., for language of treatment, second caregiver involved in treatment, caregiver race, caregiver ethnicity, caregiver level of education) to determine whether demographic variables would need to be included in subsequent logistic regression analyses. Completion of the screening paperwork did not differ significantly across any tested demographic variables. Intake attendance differed significantly by ethnicity (*χ* ^2^[1, 1111] = 4.596, *p* = 0.032). Specifically, Hispanic caregivers were less likely to attend intake than non‐Hispanic caregivers. Treatment completion differed significantly by treatment language (*χ* ^2^[2, 2289] = 9.034, *p* = 0.011; Spanish‐speaking caregivers less likely to complete treatment) and race (*χ* ^*2*^[6, 1076] = 17.997, *p* = 0.006; White families more likely to complete treatment, African American families less likely). Race and language variables were included in subsequent regression analyses predicting the outcomes with which they were significantly related. Ethnicity, however, was excluded because it significantly differed between recruitment/referral sources as well as outcomes (i.e., some of the recruitment/referral sources *only* referred Hispanic caregivers, so ethnicity within these recruitment/referral sources did not vary, making it impossible to estimate the model due to this complete covariance; Vatcheva et al. 2016), and therefore obfuscated observed differences between recruitment/referral sources. Limitations of leaving ethnicity out of these analyses are addressed in the discussion.

### 4.2 Logistic Regressions

Among families who contacted the PCIT program, most were more likely to complete the screening paperwork, except for those referred via community outreach (OR = 0.884, 95% CI [0.625, 1.252], *p* = 0.488) or schools (OR = 1.422, 95% CI [0.995, 2.034], *p* = 0.054), who were no more likely to complete than not to complete. The highest likelihood of completing the screening paperwork was observed among families referred from wraparound neighborhood services (OR = 7.089, 95% CI [3.224, 15.587], *p* < 0.001), social media (OR = 3.405, 95% CI [1.696, 6.834], *p* < 0.001), and those who were previously enrolled (OR = 3.304, 95% CI [1.114, 9.801], *p* = 0.031; see Table 4). Other referral sources were also more likely to complete than not, including pediatric primary care (OR = 2.242, 95% CI [1.842, 2.729], *p* < 0.001), word‐of‐mouth (OR = 2.295, 95% CI [1.717, 3.068], *p* < 0.001), behavioral health (OR = 2.105, 95% CI [1.574, 2.817], *p* < 0.001), Google (OR = 1.848, 95% CI [1.340, 2.548], *p* < 0.001), physician specialists (OR = 1.608, 95% CI [1.173, 2.204], *p* = 0.003), early intervention (OR = 2.987, 95% CI [1.979, 4.510], *p* < 0.001), allied health professionals (OR = 2.128, 95% CI [1.317, 3.437], *p* = 0.002), and disability advocacy organizations (OR = 1.902, 95% CI [1.190, 3.039], *p* = 0.007).

**Table 4:** Logistic regression predicting completion of screening paperwork.

Referral source	Odds Ratio	*p*	Confidence Interval
Pediatric primary care	2.242	< 0.001	1.842, 2.729
Word‐of‐mouth (friend referred)	2.295	< 0.001	1.717, 3.068
Behavioral health	2.105	< 0.001	1.574, 2.817
Google	1.848	< 0.001	1.340, 2.548
Physician specialists	1.608	0.003	1.173, 2.204
Early intervention	2.987	< 0.001	1.979, 4.510
School‐based services	1.422	0.054	0.995, 2.034
Community outreach	.884	0.488	0.625, 1.252
Allied health professionals	2.128	0.002	1.317, 3.437
Disability advocacy organizations	1.902	0.007	1.190, 3.039
Wraparound neighborhood services	7.089	< 0.001	3.224, 15.587
Social media	3.405	< 0.001	1.696, 6.834
Previously enrolled	3.304	0.031	1.114, 9.801
COVID	1.513	< 0.001	1.274, 1.798

*Note:* Reference variable for dummy coding referral sources was insurance company‐referred. *N* = 1658.

Families were also more likely to complete the screening paperwork after COVID‐19 than families were before COVID‐19 (OR = 1.513, 95% CI [1.274, 1.798], *p* < 0.001).

Then, we examined the likelihood of families attending the intake session if their child was eligible for services. Only families whose children tested as eligible for services were included in this analysis. Families from nearly all recruitment/referral sources were more likely to attend than not attend intake, except for those who had been previously enrolled in PCIT. The recruitment/referral sources that conferred the highest likelihood of attending intake were behavioral health (OR = 5.652, 95% CI [3.554, 8.988], *p* < 0.001), disability advocacy organizations (OR = 4.758, 95% CI [2.175, 10.410], *p* < 0.001), and physician specialists (OR = 4.048, 95% CI [2.505, 6.541], *p* < 0.001; see Table 5). Families were also more likely to attend the intake session after COVID‐19 than prior (OR = 3.653, 95% CI [2.267, 5.886], *p* < 0.001).

**Table 5:** Logistic regression analysis predicting attendance at intake.

Referral source	Odds ratio	*p*	95% Confidence interval
Pediatric primary care	3.527	< 0.001	2.658, 4.680
Word‐of‐mouth (friend referred)	3.484	< 0.001	2.346, 5.173
Behavioral health	5.652	< 0.001	3.554, 8.988
Google	3.653	< 0.001	2.267, 5.886
Physician specialists	4.048	< 0.001	2.505, 6.541
Early intervention	3.633	< 0.001	2.130, 6.196
School services	3.968	< 0.001	2.169, 7.261
Community outreach	3.197	< 0.001	1.685, 6.067
Allied health professionals	2.120	0.017	1.147, 3.920
Disability advocacy organizations	4.758	< 0.001	2.175, 10.410
Wraparound neighborhood services	2.824	< 0.001	1.557, 5.124
Social media	2.858	0.004	1.387, 5.891
Previously enrolled	1.187	0.765	0.387, 3.636
COVID	3.653	< 0.001	2.267, 5.886

*Note:* This analysis selected for the families whose children were eligible to receive services. Reference variable for dummy coding referral sources was insurance company‐referred *N* = 981.

Lastly, for families who attended the first intake session, we then examined the likelihood of completing treatment. Only five predictors emerged as significant. During COVID‐19, families were more likely to complete treatment than before COVID‐19 (OR = 1.525, 95% CI [1.162, 2.002], *p* = 0.002). Further, families' likelihood of completing treatment significantly increased if they were referred by their pediatric primary care (OR = 1.810, 95% CI [1.347, 2.431], *p* < 0.001), behavioral health (OR = 2.032, 95% CI [1.329, 3.108], *p* < 0.001), Google (OR = 2.054, 95% CI [1.219, 3.460], *p* = 0.007), or word of mouth (OR = 2.017, 95% CI [1.301, 3.127], *p* = 0.002). Given that treatment completion rates differed by treatment language and race, these were initially entered into the regression, but as they were not significant in the full model, they were dropped from the overall analyses (see Table 6). They were also tested as moderators of the referral sources, but none of the moderators emerged as significant so they were also excluded from final analyses.

**Table 6:** Logistic regression analysis predicting treatment completion.

Referral source	Odds ratio	*p*	95% Confidence interval
Pediatric primary care	1.810	< 0.001	1.347, 2.431
Word‐of‐mouth (friend referred)	2.017	0.002	1.301, 3.127
Behavioral health	2.032	0.001	1.329, 3.108
Google	2.054	0.007	1.219, 3.460
Physician specialists	1.106	0.687	0.678, 1.802
Early Intervention	1.252	0.416	0.728, 2.153
School services	1.200	0.542	0.668, 2.153
Community outreach	1.455	0.290	0.726, 2.916
Allied health professionals	1.407	0.377	0.660, 3.001
Disability advocacy organizations	1.726	0.164	0.801, 3.719
Wraparound neighborhood services	0.704	0.287	0.369, 1.343
Social media	1.749	0.198	0.747, 4.098
Previously enrolled	1.967	0.414	0.388, 9.973
COVID	1.525	0.002	1.162, 2.002

*Note:* This analysis only included the families who attended the first intake session. Reference variable for dummy coding referral sources was insurance company‐referred *N* = 630.

## 5 Discussion

### 5.1 Summary and Strengths

Behavioral health programs invest endless time and resources establishing referral connections, often without evidence of their effectiveness. This study extends prior research by examining specific recruitment/referral sources in recruiting and retaining predominantly disadvantaged and marginalized families, providing insights into effective strategies for reaching those most in need of mental health services.

### 5.2 Completing the Screening Paperwork

Overall, most families who contacted the PCIT program were more likely to complete their paperwork than not. The recruitment/referral source with the highest likelihood of completing screening paperwork was the Wraparound Community Services. As a community agency embedded in a nearby largely Hispanic neighborhood working as a liaison for clinic recruitment, this finding reflects previous literature emphasizing the importance of community liaisons for treatment engagement, and underscores the importance of strong community partnerships to develop patient trust in new interventions (Schoeppe et al. 2014). This finding is also largely due to the nature of the partnership. Specifically, PCIT services were embedded within this community agency, with community health workers routinely assisting families in completing the necessary paperwork and thus decreasing the family's barriers to completing screening paperwork. However, this partnership's impact diminished when it came to families attending intake and completing treatment, indicating that some additional support may be needed past the completion of the screening paperwork. Such support may take the form of additional learning and skill practice between sessions with community health workers to improve treatment continuation and adherence (Davis et al. 2022; Garcia et al. 2023).

The two sources associated with the next highest likelihoods of completing the screening paperwork were those previously enrolled and social media. It is possible that those previously enrolled were familiar with the process of completing the paperwork and therefore less deterred by the quantity of questions to complete. Similarly, those referred through social media likely followed the public link to complete the paperwork, which likely expedited the process. Those previously enrolled also had pre‐existing trust in this PCIT clinic, having received services here before. This is consistent with prior literature describing institutional trust as an important factor in recruitment for services (Schoeppe et al. 2014). Social media has demonstrated promise in past studies as a recruitment strategy (e.g., Archer‐Kuhn et al. 2022), and researchers have posited different reasons why this recruitment source is effective. Some describe the sheer number of people who access social media each day, which increases the visibility of the services being offered (Oesterle et al. 2018). Given the social connections and resources available through social media, it may also serve as a modern‐day strategy for recruitment/referral to occur via “word of mouth”—in other words, instead of speaking directly to friends to ask for recommendations for mental health support, those seeking services connect with social networks that they trust via social media. This connection through trusted sources that are perceived as close to the referring partner aligns with the theory of social proximity, in which families trust recruitment/referral sources more when they are trusted sources of information (Valente 2010).

### 5.3 Attending Intake

Families from most recruitment/referral sources who were eligible for treatment were more likely than not to attend the intake session, with the exception of families who were previously enrolled in PCIT. When families contact the clinic to re‐enroll, often their previous therapist guides them through resuming practicing the skills taught in treatment. Sometimes, if the family resumes these strategies, starting treatment over is not necessary again. Therefore, they may complete the paperwork but later decide that initiating another course of PCIT may not be necessary. Alternatively, families who were previously enrolled had a smaller sample size as they moved from screening to intake (see Table 1) and therefore the cell size may have been too small to detect significance for this referral source. Overall the results are promising that most eligible families from a wide range of referral/recruitment sources will attend their first intake session.

For the other recruitment/referral sources, when children were eligible for treatment they were more likely than not to attend their intake session, and that the specific recruitment/referral source played little role in determining whether they attended intake or not. This makes sense, as these caregivers likely have not learned the skills to parent their children more effectively, and continue to use ineffective methods, which exacerbate their child's already elevated disruptive behavior. Therefore, they are likely to be in continuous need of behavioral health services, and highly motivated to attend the intake session.

### 5.4 Treatment Completion

Families who attended their intake sessions were significantly more likely to complete treatment if they were referred by Pediatric Primary Care, Behavioral Health, Google, and Word‐of‐Mouth. Although different in important ways, these recruitment/referral sources had several commonalities. First, to engage with them, families had to express a desire or need for help (e.g., talking with pediatrician about behavior concerns, searching Google for these specific services). This help‐seeking intention has been consistently demonstrated as an important contributor to patients' willingness to engage with mental health services (Staffileno et al. 2017), as it indicates trust in the healthcare system, acknowledgement that there is a problem that needs to be addressed (e.g., child behavior), and that they can enact the change needed on that problem (e.g., caregivers believe changing their way of relating to the child might change behavior, rather than believing that other children, environments, or adults are directly causing the behavior). Staffileno et al. (2017) studied similar strategies for recruitment and found similarly strong effects for Facebook, clinic appointments, and Google on recruitment. Programs aiming to achieve comparable recruitment outcomes, can consider recruiting from sources that share this patient readiness for help, or work with referral partners to instill this readiness before referral (e.g., help families understand that child behavior is concerning, and that they have the power to change it in a positive direction).

The recruitment/referral sources that conferred a greater likelihood of completing treatment (i.e., Pediatric Primary Care, Behavioral Health, Google, and Word of Mouth) were also more likely to have families who completed their initial screening paperwork and attended their intake sessions. Engaging with these referral/recruitment sources increased family engagement from initial clinical contact to treatment completion. Therefore, these recruitment/referral sources were effective in both recruiting and retaining families for the PCIT program. These findings are notable, as much of the treatment literature typically focuses on either recruitment *or* retention. However, both are equally important to successfully reaching families in the community and closing the mental health service gap. If family behavioral health clinics are looking for recruitment/referral sources that maximize both recruitment and retention, their time and money might be best spent in Pediatric Primary Care, Behavioral Health, Google, and Word of Mouth arenas.

### 5.5 Effects of COVID‐19

In all analyses, COVID‐19 emerged as a significant predictor. During COVID‐19, families were more likely to complete screening paperwork, although this may be influenced by a change in clinic screening practices, where around the onset of COVID‐19 the clinic introduced a public screening link that families could access at any time (see Screening for more detail).

Families were also more likely to attend intake during COVID‐19. Across the world, families were experiencing more child emotional and behavioral difficulties during COVID‐19, which may have contributed, as more severe disruptive behavior tends to lead to a greater likelihood of following through with treatment (Winslow et al. 2016). Additionally, although telehealth was available in a limited capacity before COVID‐19, every case in this clinic transitioned to telehealth in March 2020. Telehealth serves to remove barriers to treatment, increasing the likelihood of families attending intake and then completing treatment (Garcia et al. 2021; Peskin et al. 2024).

Pre‐COVID‐19, African American families were less likely to complete treatment, consistent with other studies of manualized treatments, specifically behavioral parent training (Fernandez et al. 2011; Jent et al. 2024). However, this disparity was not observed during COVID‐19. While this finding could reflect the smaller number of African American families who engaged in services during COVID‐19, it could also suggest that telehealth delivery might have mitigated barriers to treatment completion, something reflected by other telehealth studies (Comer et al. 2017).

Notably, COVID‐19 dramatically changed the landscape of recruitment/referral to mental health services. A greater number of families were referred, screened, and attended intake from March 2020 to 2022 than before COVID‐19 (i.e., 2018 to March 2020). As a multitude of others have shown (World Health Organization [WHO] 2020), families experienced a higher need and drive for mental health services as a result of COVID‐19 and its sequelae. The option to receive services via telehealth likely also contributed to the ease of attending intake, as transportation, childcare, and scheduling barriers were decreased as a result (Koerting et al. 2013).

This study represents some of the first analyses of recruitment during the COVID‐19 pandemic. Families during the pandemic experienced significant changes in income, lifestyle (e.g., working from home, decrease in leisure activities due to business closures), and stress level. These changes have reverberated for years to catalyze one of the largest mental health epidemics on record (WHO 2020). Therefore, understanding how to effectively reach, recruit, and retain children and families in need of mental health services is particularly vital at this time.

### 5.6 Limitations and Future Directions

This study's conclusions should be considered alongside the identified limitations that may have affected the results. First, the accuracy of family reports of recruitment/referral sources varied between families. Families did not always immediately recall from where they were referred, sometimes reporting one recruitment/referral source at their initial eligibility screening, but then reporting a different source on intake forms. The data utilized for this study were uniformly obtained from the initial eligibility screening, unless these data were missing, in which case the information was gathered from the intake forms. At times, families were unable to recall any specific referral source, limiting the ability to know the full scope of recruitment efforts. Future research to address this limitation could develop unique identifiers for flyers or electronic links to the screening paperwork which can inform where the family may have learned about the program.

An additional limitation includes the lack of a standardized referral process between different community‐provider‐based referral partners. As previously described, the referral process differed across agencies/providers. One key difference is variability in the relationship with each agency, ranging from a well‐established working connection with embedded PCIT clinics to more informal referral relationships involving distribution of flyers/forms. Agencies also differed in their referral systems (e.g., completing their own referral forms, making phone calls to the PCIT screening line, completing a PCIT referral form, etc.). Future studies could standardize the referral process to determine whether the process itself or the specific partner contributed to differences in rates of referral/screening completion, and so forth. Standardization could include training key staff members in these procedures, and/or designating one individual to handle referrals. Teasing apart differences between recruitment/referral sources may also benefit from further studies that examine the effectiveness of each method of referral in addition to the source. Of note, standardization of referral procedures will ultimately sacrifice some of the external/ecological validity of understanding the referral process. Future research may also benefit from an examination of whether different families benefit from different approaches to referral and recruitment. For example, some families may be more likely to follow a Google link to find a provider, and some families may be more likely to take advice from a friend or their pediatrician. Analyzing these family‐level differences may allow mental health agencies and organizations to be more tailored in their approach to recruitment.

Another limitation of this study includes changes to the screening/intake process in this PCIT program which may have affected success rates of recruitment and retention. Throughout the 5 years represented in this study, the screening and data team adjusted the screening process several times to improve families' ability to complete screening paperwork and attend intake sessions, with the goal of improving access to services. It is possible that these different interventions (e.g., shortening the length of the screening paperwork, creating a public link for forms) may have impacted outcomes measured here like intake attendance. However, since these interventions were uniformly implemented at specific timepoints and applied equally across recruitment/referral sources, they are unlikely to explain the results observed in this study.

Another limitation of this study includes the exclusion of ethnicity as a predictor despite its significant correlation with intake attendance. As mentioned above, although attendance at intake differed for Hispanic families, ethnicity was not included as a predictor due to its high correlation with several of the recruitment/referral sources. However, this remains a limitation that merits further consideration, as demographic differences in intake attendance could indicate a clinic's difficulties reaching more hard‐to‐reach populations for treatment. Future studies would need to recruit more heterogenous samples from each recruitment/referral source to enable more effective disentangling of these ethnicity results.

A further limitation of this study is that time on the waitlist was not included, as it could not be identified with the available data. In some cases, families who are on a waitlist for services for too long may decrease their motivation for services or find services elsewhere, which would impact attendance at intake. In this case, recruitment existed continuously and was never paused, so it was unlikely that the waitlist disproportionately affected one recruitment/referral source more than another, but this is difficult to ascertain with the data available and would be a worthwhile avenue of future research.

An additional limitation includes the lack of transparency about other agencies' instances of referring to this PCIT program. Due to the sheer number of agencies referring to this PCIT program, it would be logistically difficult if not impossible to track every child who was referred for services to determine whether or not they followed through with that referral. Compounding this difficulty is the need for privacy (i.e., HIPAA regulations) that patients have when seeking mental health treatment; many agencies would be unable to share identifying information about the families they referred to PCIT, and most community partners lack the staff to coordinate such communications. In addition, some common referral sources, like Google and word‐of‐mouth would be impossible to track, thus making the comparison between referral sources impossible as well. However, this lack of understanding of how many participants were originally told about the program only allows analyses here to include families who actually contacted the clinic, thus limiting our understanding of the effectiveness of all of our community referral pathways.

While this study highlights the general effectiveness of multiple referral sources, it does not address the question of ‘what works for who.’ Specifically, marginalized families may face unique barriers that require additional supports from their referral sources to ensure successful engagement. Exploratory moderation analyses to explore differential effects of referral sources across subgroups did not produce any significant moderation findings, but this could also be attributed to very small cell sizes for interaction terms rather than true nonsignificance. Future research should prioritize this line of inquiry, with particular attention to how referral sources can better support harder‐to‐reach families and mitigate systemic barriers to accessing care. Understanding these dynamics is essential for tailoring recruitment and retention strategies to meet the needs of diverse populations.

Although the PCIT program in this study has several community‐embedded clinics throughout the county, the primary clinic is located within an academic medical center, and its lead trainers and supervisors are university faculty. It is possible that being housed within a university may impact how medical and behavioral health professionals in the community view the PCIT program (e.g., more likely to trust the program to refer families). Therefore, it will be important for agencies to use this study to see how they can analyze their own recruitment/referral sources, and not to extrapolate that the same recruitment/referral sources here will work just as well in another mental health setting with a different population.

## 6 Conclusion

This study marks one of the first attempts to examine sources of referral across recruitment, intake attendance, and retention through treatment. Many studies examine either recruitment *or* retention, and it is clear from these results that the findings of where to exert more effort will be vastly different depending on the time point. Findings here reflect the importance of the community/cultural liaison and other recruitment/referral sources families trust in recruiting participants for mental health services. Additionally, family desire for help appeared to be key to completing treatment. Word of mouth, Google, Pediatric Primary Care, and Behavioral Health were highly effective recruitment/referral sources, demonstrating that help‐seeking intention continues to be an important factor in engagement in mental health services. Consequently, it may be beneficial for mental health service providers to recruit from sources that identify or instill this readiness in families before referral.

## Author Contributions

**Abigail Peskin:** Conceptualization, methodology, formal analysis, writing – original draft and subsequent edits. **William Andrew Rothenberg:** Conceptualization, methodology, supervision of formal analysis, validation, writing – review and editing. **Camille Perez:** Conceptualization, writing – original draft and review and editing. **Cindy Sobalvarro:** Conceptualization, writing – original draft and review and editing. **Eileen Davis:** Supervision, project administration, critical review, writing – review and editing. **Elana Mansoor:** review and revision of the manuscript. **Jason Jent:** Supervision, project administration, critical review, writing – review and editing, funding acquisition, investigation. **Dainelys Garcia:** Supervision, project administration, critical review, writing – review and editing, funding acquisition, data curation, investigation.

## Ethics Statement

All study procedures were undertaken in accordance with the APA Ethics Code and declaration of Helsinki. All parents consented and all children assented to participate in this study. Each aspect of this study received IRB approval from the University of Miami Miller School of Medicine IRB.

### 1 Peer Review

The peer review history for this article is available at https://www.webofscience.com/api/gateway/wos/peer-review/10.1002/jcop.70019.

## Data Availability

Data can be made available by the first author upon reasonable request.
